# Herbivory as an important selective force in the evolution of floral traits and pollinator shifts

**DOI:** 10.1093/aobpla/plw088

**Published:** 2016-12-22

**Authors:** Tania Jogesh, Rick P. Overson, Robert A. Raguso, Krissa A. Skogen

**Affiliations:** 1 Plant Science and Conservation, Chicago Botanic Garden, 1000 Lake Cook Road Glencoe, IL 60022, USA; 2 Department of Neurobiology and Behavior, Cornell University, 215 Tower Rd, Ithaca, NY 14853, USA

**Keywords:** Bee, Calylophus, diurnal pollination, florivore, hawkmoth, herbivore, Hyles, Mompha, nocturnal pollination, Oenothera, Onagraceae, pollinator shifts

## Abstract

Floral trait evolution is frequently attributed to pollinator-mediated selection but herbivores can play a key role in shaping plant reproductive biology. Here we examine the role of florivores in driving floral trait evolution and pollinator shifts in a recently radiated clade of flowering plants, *Oenothera* sect. *Calylophus*. We compare florivory by a specialist, internal feeder, *Mompha*, on closely related hawkmoth- and bee-pollinated species and document variation in damage based on floral traits within sites, species and among species. Our results show that flowers with longer floral tubes and decreased floral flare have increased *Mompha* damage. Bee-pollinated flowers, which have substantially smaller floral tubes, experience on average 13% less *Mompha* florivory than do hawkmoth-pollinated flowers. The positive association between tube length and *Mompha* damage is evident even within sites of some species, suggesting that *Mompha* can drive trait differentiation at microevolutionary scales. Given that there are at least two independent shifts from hawkmoth to bee pollination in this clade, florivore-mediated selection on floral traits may have played an important role in facilitating morphological changes associated with transitions from hawkmoth to bee pollination.

## Teaser

The diversity of floral forms in angiosperms is frequently attributed to pollinators but herbivores can be just as important. Here we examine the role of a flower-feeding caterpillar, Mompha, in driving floral trait evolution and subsequent pollinator-shifts in a recently radiated clade of flowering plants, Oenothera sect. Calylophus (Onagraceae). Longer-tubed, hawkmoth flowers had substantially greater damage compared to smaller bee-pollinated flowers. Mompha's preference for longer-tubed flowers, which is evident even within populations of some species, may have played an important role in driving the morphological changes associated with shifts from hawkmoth to bee pollination.

## Introduction

Herbivores have been cited as an important evolutionary force in the diversification of angiosperms (Futuyma and Agrawal 2009). Since Ehrlich and Raven’s seminal paper on escape-and-radiate coevolution (Ehrlich and Raven 1964), numerous studies have established the importance of herbivores for the evolution of plants at micro- and macroevolutionary scales (Agrawal *et al.* 2012; Ågren *et al.* 2013; Coley and Kursar 2014; Becerra 2015). Yet the evidence for herbivore-mediated diversification in plants remains elusive. A putative mechanism by which herbivores might influence diversification in plants is through the alteration of plant-pollinator interactions (Janz 2011; Althoff *et al.* 2014; Johnson *et al.* 2015; Marquis *et al.* 2016).

It is widely recognized that adaptation to pollinators is responsible for the incredible diversity of floral forms in angiosperms (Fenster *et al.* 2004). Indeed, floral morphology, color pigments and scent compounds converge based on pollinator functional groups in distant and unrelated species (Schiestl and Johnson 2013). Many studies have examined floral adaptations to pollinators but we have only begun to appreciate the role of non-pollinator interactions in shaping plant reproductive biology (Strauss and Whittall 2006; Armbruster *et al.* 2009; Johnson *et al.* 2014). One such interaction, herbivory, can impose strong selection on floral colour (Irwin *et al.* 2003; Frey 2004; Carlson and Holsinger, 2012), floral scent (Gross *et al.* 2016), morphology (Galen and Cuba 2001; Sun *et al.* 2016), flowering phenology (Brody 1997) and even mating systems (Kariyat *et al.* 2013; Carr and Eubanks 2014), traits typically attributed to selection by pollinators. In a classic example, Galen and Cuba (2001) showed that both bumblebee pollinators and nectar-thieving ants preferentially visit flowers with larger floral flares in *Polemonium viscosum* (Polemoniaceae). Floral flare mediates access to nectar and at lower elevations, where ants are abundant, flowers are under selection to reduce flare and limit nectar robbery (Galen 1999). Herbivory can also influence mating system evolution; selfing and inbreeding in some *Solanum* species can impair plant defenses resulting in high rates of herbivory, which should favour outcrossing (Kariyat *et al.* 2011). Using phylogenetic comparative methods, Johnson *et al.* (2009) showed that sexually reproducing taxa are better defended in comparison to their functionally asexual relatives in evening primroses (Onagraceae). Similarly, self-compatible species in the Solanaceae invest more in induced defenses (Campbell and Kessler 2013). Together, these studies suggest that antagonistic interactions are closely tied to plant reproduction even at longer temporal scales.

Conversely, pollinator-mediated selection on floral traits can have important implications for plant-herbivore interactions. In *Dalechampia* (Euphorbiaceae) vines, pollinator-mediated selection of key floral traits (like the production of resin rewards) can determine the subsequent evolution of plant defenses (Armbruster *et al.* 2009). Shifts in floral traits can alter the feeding preferences of herbivores; for example, anthocyanin plays a key role in plant defense (Johnson *et al.* 2008) and pollinator-mediated shifts in floral colour can influence defense phenotypes. Alternatively, by imposing strong selection on floral traits, herbivores have the potential to influence speciation via pollinator shifts. In a striking example, Kessler *et al.* (2010) demonstrate that herbivory can shift pollinator preference within the lifetime of a plant. For *Nicotiana attenuata*, attracting adult *Manduca* hawkmoths to flowers comes with the disadvantage of associated *Manduca* larval herbivory. In years where larval herbivory was especially detrimental, plants switched to morning anthesis, reduced benzyl acetone floral scent emissions and were preferentially pollinated by hummingbirds over hawkmoths. Plant-pollinator and plant-herbivore interactions are not mutually exclusive and often, key floral traits that determine pollinator preference and efficacy, also predict herbivory, including plant size (Gomez and Olivieri 2003), flower size, floral rewards (Mothershead and Marquis 2000) and floral scent (Galen *et al.* 2011). Given that these interactions are correlated, herbivores can reduce the strength of pollinator-mediated selection on floral traits and vice versa. Herbivory (and florivory) is ubiquitous across angiosperms but only a handful of studies have examined the role of herbivores in driving floral trait-evolution over longer evolutionary time scales (but see Armbruster *et al.* 2009; Johnson *et al.* 2009; Adler *et al.* 2012). Further, numerous recent reviews (for e.g. Marquis *et al.* 2016) suggest that herbivores influence angiosperm diversification via alterations to plant mutualistic networks but to date, no studies outline a process by which herbivores can influence pollinator-shifts in a group of closely related flowering plants.

**Figure 1:**
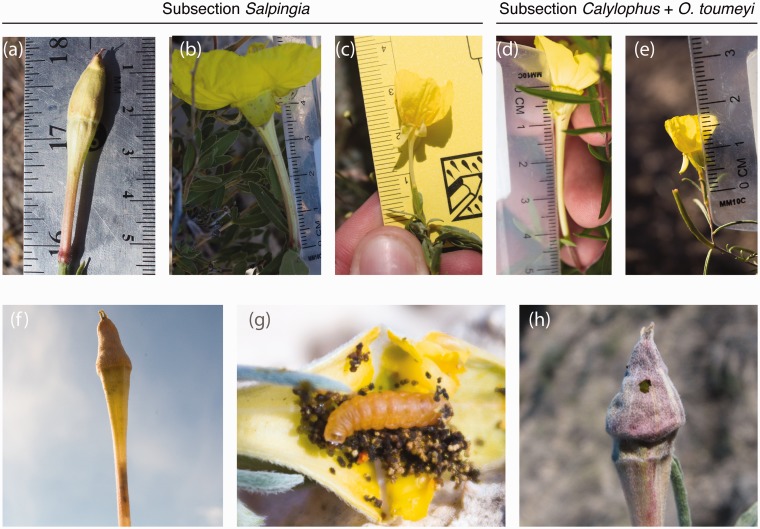
Floral tube length of (a) *O. lavandulifolia*, (b) *O. hartwegii* subsp. *pubescens* (c) *O. tubicula* subsp. *tubicula*, (d) *O. toumeyi* (e) *O. gayleana* and (f-h) evidence of *Mompha* damage on floral buds.

In this study, we compare herbivory in closely related hawkmoth- and bee-pollinated species in *Oenothera* sect. *Calylophus*, a monophyletic group that presents an ideal system in which to examine the association between herbivory and pollination in a recently radiated clade of flowering plants. First, hawkmoth pollination is the ancestral condition for this group (Wagner *et al.* 2007) with at least two independent shifts to bee pollination (Towner 1977, Cooper 2016). Hawkmoth- and bee-pollinated taxa have distinct floral morphologies (Fig. 1) and are differentiated by the length of the floral nectar tube. Hawkmoth- and bee-pollinated taxa also differ in the timing of anthesis with bee-pollinated taxa opening in the early morning and hawkmoth-pollinated taxa opening in the evening. Hawkmoth- and bee-pollinated taxa in *Oenothera* sect. *Calylophus* are also broadly sympatric and commonly flower synchronously. Second, nearly all species are colonized by small moths in the genus *Mompha*, the caterpillars of which are internal feeders of Onagraceae that create leaf mines, stem galls, or feed within flowers and fruits. Within *Oenothera* Sect. *Calylophus, Mompha* feed primarily on floral structures within unopened buds, resulting in a substantial fitness cost, as these buds do not open (Fig. 1). This group of *Mompha* putatively belongs to a single species complex, *Mompha pecosella* (Bruzzese 2016). *Mompha* larvae collected on sympatric species in western Texas and southeastern New Mexico are genetically very similar suggesting that the same florivore infests nearly all plant species within *Oenothera* Sect. *Calylophus* (Bruzzese 2016).

Because *Mompha* are internal feeders within floral tissues in this group, florivory should be tightly linked to floral morphology. Further, the crepuscular behavior of *Mompha* adults suggests that oviposition occurs in the evening, making vespertine, hawkmoth-pollinated plants more apparent and vulnerable to *Mompha* florivory compared to bee-pollinated taxa. Due to the fitness cost associated with *Mompha* florivory, we hypothesize that these florivores impose strong selection on floral traits and the timing of anthesis, which has played an important role in driving morphological changes associated with shifts from hawkmoth to bee pollination. Alternatively, it is possible that floral trait diversification owing to pollinator-mediated selection has had important implications for *Mompha* florivory. We predict that (1) *Mompha* florivory (which results in a direct loss in plant fitness) differs between hawkmoth and bee-pollinated species and (2) *Mompha* florivory is associated with floral traits that differentiate hawkmoth- and bee-pollinated plants. Even though it is challenging to predict past selective pressures from current evolutionary end points, by comparing florivory and pollination in a group of closely related species, we can evaluate the relative roles of these interactions in shaping the evolution of this group.

## Methods

### Study system

#### Oenothera *sect.* Calylophus

*Oenothera* sect. *Calylophus* has seven currently recognized species (13 taxa) in two morphologically distinct subsections, subsect. *Salpingia* (*O. hartwegii, O. lavandulifolia, O. toumeyi* and *O. tubicula*) and subsect. *Calylophus* (*O. capillifolia, O. gayleana*, and *O. serrulata*; Towner 1977; Turner and Moore 2014). Molecular phylogenetic analyses show strong support for the two subsections with the exception of *O. toumeyi*, which is not in subsect. *Salpingia* but is sister to subsect. *Calylophus* (Cooper 2016). Many taxa are geographically and morphologically variable, making species boundaries unclear for some taxa. Based on phenotypic variation, five subspecies have been described for *O. hartwegii* (*O. hartwegii* subsp. *pubescens, O. hartwegii* subsp. *filifolia, O. hartwegii* subsp. *hartwegii, O. hartwegii* subsp. *maccartii, O. hartwegii* subsp. *fendleri)*, two for *O. tubicula* (*O. tubicula* subsp. *tubicula* and *O. tubicula* subsp. *strigulosa*) and two for *O. capillifolia* (*O. capillifolia* subsp. *capillifolia* and *O. capillifolia* subsp. *berlandieri)*.

The majority of the diversity in this group of plants is centred in the southwestern United States and in northern Mexico (Towner 1977; Turner and Moore 2014), the exception being *O. serrulata*, which also occurs throughout the Great Plains and into adjacent southern Canada. All species are perennial herbs or subshrubs, usually found growing in calcareous soils of the Chihuahuan Desert. *Oenothera hartwegii* subsp. *filifolia* and *O. gayleana* are endemic to gypsum soils (Turner and Moore 2014). All species are self-incompatible and rely on insect pollination for reproduction, with the exception of *O. serrulata*, which exhibits permanent translocation heterozygosity and is therefore functionally asexual (Towner 1977). Although the floral morphology of the majority of the taxa in section *Calylophus* is consistent with expectations of hawkmoth pollination, three species, *O. tubicula*, *O. capillifolia* and *O. gayleana*, have floral morphologies reflective of bee pollination with smaller flowers, shorter floral tubes, wider floral flares, distinct UV patterns, reduced floral scent and little to no nectar (Fig. 1). The flowers of the hawkmoth-pollinated taxa have longer floral tubes (up to 70 mm), narrower floral flares and are scented in some cases. Hawkmoth flowers also show UV reflectance patterns but not as prominently as those of bee-pollinated taxa (Towner 1977). All species have yellow petals that fade to orange or red. Anthesis in bee-pollinated flowers is early in the morning, prior to sunrise (∼ 04:00 - 05:00 hrs) whereas anthesis in hawkmoth flowers is generally in the evening (∼18:00 hrs.). Flowers can remain open for 36-60 hours following anthesis.

#### Mompha sp

*Mompha* (order: Lepidoptera, superfamily: Gelechioidea, family: Momphidae) is a genus of small herbivores with a global distribution and approximately 40 species in North America (Powell and Opler 2009). The larvae of *Mompha* are almost exclusively internal plant feeders, feeding within leaves, stems, roots, flowers and fruits. The majority of the North American *Mompha* specialize on Onagraceae with a few species feeding on Lythraceae, Cistaceae and Rubiaceae (Powell and Opler 2009). *Oenothera* sect. *Calylophus* is primarily colonized by flower/bud borers, which feed on the reproductive parts (style, filaments) and petals of unopened buds. The caterpillars feed internally for the duration of their lifetime and occasionally the hypanthium of infected buds swells to create a gall-like feeding enclosure. Similar gall-like structures have been observed on *Cuphea* (Lythraceae) (Graham 1995). Prior to pupation, the caterpillars chew a hole through the bud and drop to the ground (Fig. 1H). The adults are active at dusk and have been observed visiting open flowers (Artz *et al.* 2010), but little is known about the mating biology and oviposition behavior of adult moths. *Mompha* on *Oenothera* Sect. *Calylophus* are likely multivoltine owing the prolonged flowering phenology of their hosts and the moths presumably overwinter as pupae or adults.

### Data collection

In this study, we examined floral traits, florivory and pollinator visitation in five taxa across their geographic range: *O. hartwegii* ssp. *pubescens*, *O. lavandulifolia*, *O. toumeyi, O. tubicula* ssp. *tubicula* and *O. gayleana.* We identified four to five locations per species that were representative of their range, for a total of 19 populations (Table 1). We were only able to collect data from two populations of *O. toumeyi*, as it is limited to three sky-island mountain ranges in southern Arizona (Chiricahuas, Huachucas and Santa Ritas) within the continental United States. Voucher specimens from each location were deposited at the Nancy Rich Poole Herbarium (Chicago Botanic Garden, CHIC) and the Smithsonian Institute (US). At each location, we measured floral traits and evaluated florivory on 15 to 30 individual plants and recorded pollinator visits on 1 to 19 open flowers per plant for an average of 6 plants per site at all 19 populations in this study. Floral trait and florivory data were collected on the same individuals at the same time for all populations except DCW (*O. lavandulifolia*), the data for which were not included in combined analyses of floral traits and florivory. Flowers were excised from the plants at the base of the ovary and the following morphological traits were measured on one flower per plant: corolla diameter, hypanthium length, floral flare and herkogamy (stigma-anther separation) to the nearest 0.01 mm using digital calipers. Corolla diameter was measured along two of the longest petal axes, perpendicular to the nectar tube. Hypanthium length was measured from the top of the ovary to the point of sepal insertion at the end of the hypanthium. Floral flare was measured as the diameter of the opening to the hypanthium. Style and filament lengths were measured separately. Because the filaments are adnate to the hypanthium, filament and hypanthium lengths were summed and subtracted from the style to calculate herkogamy. Nectar was collected in a 50.0 µl microcapillary tube (Drummond Microcaps, Broomall, Pennsylvania, USA) after making an incision at the base of the hypanthium with a razor. Nectar length in the microcapillary tube was used to calculate nectar volume. We then dried the flowers in silica gel and weighed them to the nearest mg to get an approximation of overall flower mass and size.

**Table 1:** Site, location and collection information for all populations sampled in this study.

Subsection	Species	Site	State	County	Latitude	Longitude	Date collected	Sample size
Salpingia	*O. lavandulifolia*	10 miles S. of Alpine	TX	Brewster	30.22089	−103.56819	7/22/15	30
		DCW	CO	Otero	37.75817	−103.61902	5/15/13	25
		Silver Creek Rd	NV	White Pine	39.13376	−114.21691	6/3/14	34
		Slickrock	CO	San Miguel	38.02745	−108.9008	5/17/14	32
		Tan Seeps	UT	Emery	39.02615	−110.69511	5/19/14	30
	*O. tubicula tubicula*	Black River Village	NM	Eddy	32.23544	−104.21759	7/5/15	20
		Box Canyon Rd	NM	Eddy	32.45642	−104.76361	9/3/14	34
		Nine Point Mesa	TX	Brewster	29.72728	−103.56577	7/20/15	30
		Picacho	NM	Lincoln	33.35138	−105.14169	7/3/15	21
		Pine Springs	TX	Culberson	31.89377	−104.81779	7/8/15	32
	*O. hartwegii pubescens*	Hwy 82 mm 55	NM	Chaves	32.89753	−105.23849	9/3/14	30
		Sierra Diablo	TX	Culberson	31.15905	−104.83454	9/9/14	30
		South of Stockton	TX	Pecos	30.74542	−102.90897	7/16/15	32
		Taiban	NM	DeBaca	34.35309	−104.00141	9/1/14	17
Calylophus	*O. gayleana*	Croton Camp	TX	Dickens	33.47678	−100.8554	7/14/15	30
		Gas Line Rd	NM	Eddy	32.03693	−104.43731	8/28/14	31
		Seven Rivers Hills	NM	Eddy	32.56243	−104.42569	7/11/15	30
		Trigg Ranch	NM	Debaca	34.15499	−104.48089	7/2/15	30
	*O. toumeyi*	Carr Canyon Rd	AZ	Cochise	31.43183	−110.28216	9/15/14	30
		Pinery Canyon Rd	AZ	Cochise	31.93941	−109.2886	9/13/14	32

*Mompha* florivory was evaluated for each sampled plant by systematically inspecting flower buds for evidence of caterpillars (or caterpillar frass) in all buds or a subset of 10 buds if a plant had more than 10 buds. Because buds infested with *Mompha* rarely open and because *Mompha* caterpillars effectively eat almost all reproductive structures (personal observation), the proportion of infested buds is indicative of direct losses of fitness to *Mompha* florivory. Therefore, we quantified *Mompha* florivory as the proportion of examined buds with evidence of *Mompha* to account for variation in sampling effort. We also recorded evidence of additional herbivores including *Hyles lineata* (Lepidoptera: Sphingidae) eggs and caterpillars, *Altica* beetles, aphids and other foliar and floral herbivores; however, these observations were not frequent enough to make meaningful comparisons across sites and species.

To confirm pollinator functional groups for each species, we conducted pollinator observations in the morning (∼7:00), afternoon (∼12:00–14:00) and in the evening (∼18:00) for 30 to 60 minutes using a combination of 3–4 video cameras and 2–5 human observers. An average of 67 flowers was observed for 118 minutes per site at all 19 populations in this study. We recorded floral visits based on insect contact with the flowers’ reproductive structures (stigma or anthers). While watching flowers, we identified most hawkmoths to species but bees were difficult to identify in the field and were categorized based on their size (small, medium, large). Total sampling effort (observer hours) varied between populations and years based on the available resources and weather conditions and visitation rates were calculated per flower per hour. For each site and species, we averaged the visitation rate per pollinator group (identified to the lowest possible taxonomic rank) to compare pollinator functional groups.

### Statistical analyses

#### Floral traits

All statistical analyses were conducted in R 3.0.1 (R Core Team 2015). To compare differences in floral traits between species, we first calculated a Bray-Curtis dissimilarity distance matrix using square-root transformed morphological traits including corolla diameter, floral flare, tube length, herkogamy, nectar volume and flower mass. Using this distance matrix we performed a non-parametric multidimensional scaling (NMDS) analysis with the package “ecodist” (Goslee and Urban 2007) to visualize floral trait differences among species. To test for differences between species, pollinator functional group (bee versus hawkmoth) and relatedness (taxonomic group: subsect. *Salpingia* or subsect. *Calylophus*), we conducted an ANOSIM (analysis of similarity) implemented in the package “vegan” (Oksanen *et al.* 2015). Similar to an analysis of variance, ANOSIM compares the dissimilarity matrix within and between groups and generates a test statistic *R*, based on a non-parametric permutation procedure (Clarke 1993). Values of R close to 1 indicate a complete separation between groups while R close to zero indicates minimal separation. We also conducted a similarity of percentages analysis (SIMPER) to determine the average contribution of each trait to overall differentiation between species.

#### Mompha florivory

We were interested in examining variation in *Mompha* florivory based on floral traits, between sites and species, and based on pollinator functional group (hawkmoth or bee). To evaluate these relationships, we ran a series of generalized linear hierarchical Bayesian models (listed in Table 2). The response variable was the log-transformed proportion of buds with *Mompha* in all models. Floral traits were standardized for ease of interpretation and pollinator functional group was coded as 0 (bee pollination) and 1 (hawkmoth pollination) prior to model fitting. Taxonomic group (subsect. *Salpingia* or subsect. *Calylophus *+* O. toumeyi*), species and site were modeled as variable intercepts.

**Table 2:** Models tested for the effect of species, site, pollination syndrome and floral morphology on the proportion of buds infected with *mompha*. N is the number of individuals plants analyzed, j is the number taxonomic groups (subsect. *Salpingia* or subsect. *Calylophus *+* O.Toumeyi*) (j = 2), k is the number of species (k = 5) and l is the number of sites in the study (l = 19). the response variable is the log (#buds examined with evidence of *mompha*).

Model number	Hierarchical variable intercepts	Predictors	Sample size	Question
1	Species [Taxonomic group]	–	*n*=504, j = 3, k = 5	What is the taxonomic variation in *Mompha* herbivory while controlling for species relatedness?
2	Site [Species [Taxonomic group]]	–	*n*=504, j = 3, k = 5, l = 19	What is the site variation in *Mompha* herbivory while controlling for species and relatedness between species?
3	Taxonomic group	Pollinator functional group	*n*=504, j = 3	Can pollinator syndrome predict *Mompha* herbivory while account for relatedness between species?
4	Taxonomic group	Morphological traits (corolla diameter, floral flare, herkogamy, nectar volume, tube length)	*n*=419, j = 3	Which morphological traits predict *Mompha* herbivory overall?
5	Species [Morphological traits]*	Morphological traits	*n*=419, k = 5	Does the magnitude and direction of the relationship between floral morphology and *Mompha* change with species?
6	Site [Morphological traits]*	Morphological traits	*n*=419, l = 19	Does the magnitude and direction of the relationship between floral morphology and *Mompha* change with site?

* interaction between slopes and intercept.

We used uninformative normal priors for all coefficients with a mean of zero and standard deviation of 100 (inverse-variance of 0.0001) and uniform priors for the standard deviation for all error terms with a range from 0 to 100 (Gelman and Hill 2007). Bayesian models were implemented in the BUGS language (Gilks *et al.*, 1994; Lunn *et al.*, 2009) using JAGS (version 3.2.0; Plummer 2003) and run in R using the rjags and coda packages (Plummer *et al.*, 2006). Posterior distributions for model parameters were estimated using Markov chain Monte Carlo (MCMC) simulations in JAGS (version 3.2.0; Plummer, 2003). We ran four MCMC chains for 100,000 iterations and supplied initial values for all parameters, which were obtained from random numbers in a normal distribution. Initial values for error terms were supplied from a uniform distribution that was constrained to be positive. The first 5,000 iterations were discarded and for all parameter estimates, we confirmed that the four chains had approximately converged by ensuring that the potential scale reduction factor R.hat was less than 1.01 for all parameter estimates (Gelman and Rubin, 1992). In models where the slopes were allowed to vary by species and sites (Models 5 and 6), the covariance matrix between intercepts and slopes was modeled using a scaled-inverse Wishart distribution (Gelman and Hill 2007). R and Jags code for all models are provided **[see Supporting Information—File S1 and S2]**.

In our first two models, we compared differences in florivory between sites and species and included taxonomic group as a variable intercept to account for relatedness among species (Table 2). The first taxonomic unit comprised of all species in subsect. *Salpingia* and the second taxonomic unit included *O. gayleana* (in subsect. *Calylophus*) and *O. toumeyi*. Even though *O. toumeyi* is not in subsect. *Calylophus* as currently defined, it is a sister to this group (Cooper 2016). In our third model, we evaluated differences in *Mompha* florivory between hawkmoth- and bee-pollinated taxa. We then examined the relationships between floral traits and *Mompha* florivory at three hierarchical levels. Model 4 predicts florivory based on floral traits across all species while controlling for *Mompha* variation between taxonomic groups. Models 5 and 6 allowed the slope parameters to vary by species (species by floral trait interaction) and site (site by floral trait interaction). Variable slope models allowed us to estimate the magnitude and the direction of the relationships between floral traits and *Mompha* florivory within each species and within each collection site.

## Results

**Figure 2:**
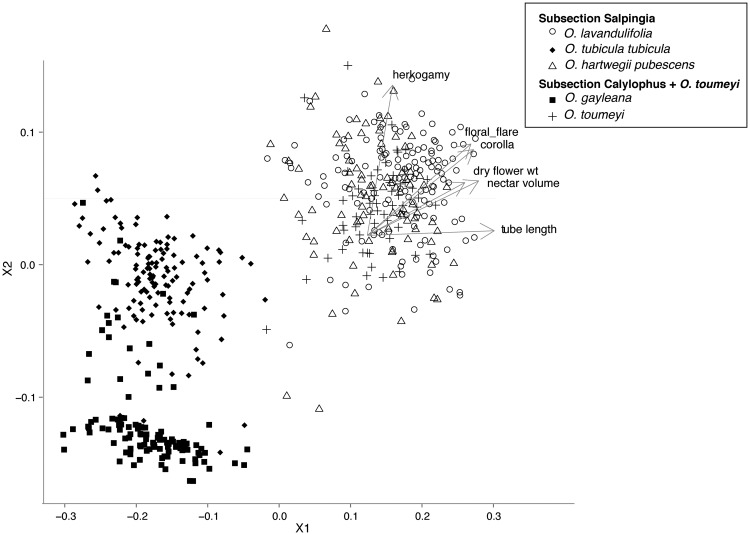
NMDS ordination plot for all analyzed individuals. Bee-pollinated species are marked by filled symbols and hawkmoth-pollinated species by open symbols.

We analyzed a total of 525 individuals from 5 species for differences in floral traits. We see a clear distinction in the morphological traits of hawkmoth- and bee-pollinated flowers with bee-pollinated taxa having smaller corollas, floral flares, floral tube lengths, lower nectar volumes and almost no herkogamy (Fig. 2). Floral traits showed near complete separation based on pollination syndrome (ANOSIM *R*  =  0.99, *P*  =  0.001) and to a lesser extent based on species (ANOSIM *R*  =  0.73, *P*  =  0.001). While the two bee-pollinated species, *O. gayleana* and *O. tubicula* subsp. *tubicula*, were strongly differentiated based on herkogamy (Fig. 2, ANOSIM *R*  =  0.62, *P*  =  0.001), hawkmoth-pollinated species were indistinguishable (ANOSIM *R*  =  0.05, *P*  =  0.008). Floral traits did not show substantial separation based on relatedness (membership to taxonomic group) (ANOSIM *R*  =  0.31, *P*  =  0.001). Tube length contributed the most to floral differentiation (SIMPER contribution 11%) and floral flare contributed the least (SIMPER contribution 0.4 %). All floral traits were strongly correlated with each other across all species, however, within species, only style length was strongly correlated to tube length (Pearson’s R > 0.80).

**Figure 3:**
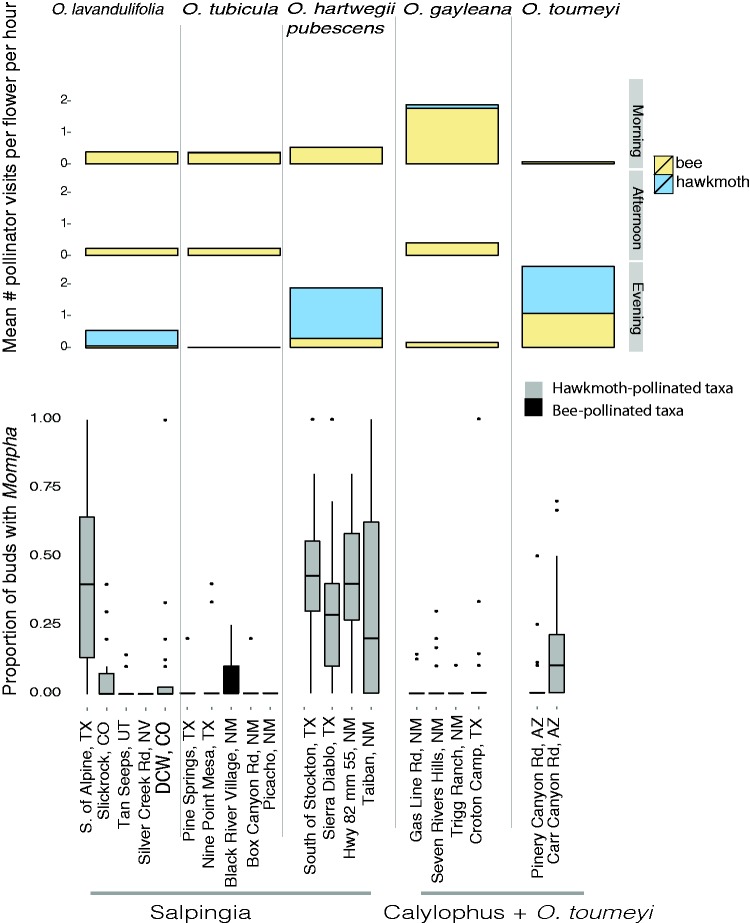
Pollinator visitation rates per species and the proportion of buds with *Mompha* per site.

**Figure 4:**
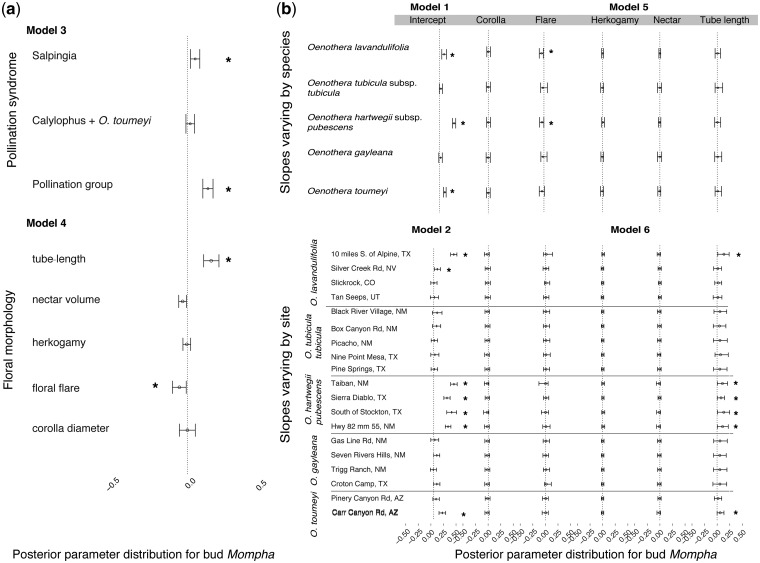
Median posterior coefficients (points) and 95% credible intervals (CI) for intercepts and slopes predicting *Mompha* bud loss for all Models listed in Table 1. CIs for intercepts and slopes not overlapping with 0 are denoted by *.

Florivory varied significantly between species and sites and hawkmoth-pollinated species (*O. lavandulifolia*, *O. hartwegii* subsp. *pubescens* and *O. toumeyi*) had considerably greater levels of florivory (Figs 3 and 4). Overall, hawkmoth-pollinated plants had a 13% increase in *Mompha* damage compared to bee-pollinated plants (Model 3, Fig. 4 credible interval 0.08, 0.15). *Oenothera hartwegii* subsp. *pubescens* had the highest *Mompha* florivory with on average 30% more buds with *Mompha* (Model 1 Fig. 4 credible interval 0.23, 0.30), followed by *O. lavandulifolia* (Model 1 Fig. 4 credible interval 0.07, 0.12), and *O. toumeyi* (Model 1 Fig. 4 credible interval 0.04, 0.12). Flowers with longer floral tubes and smaller floral flares had more *Mompha-*infested buds (Fig. 4A). A 18.2 mm average increase in tube length was associated with a 13% increase in the proportion of buds with *Mompha* and a 2.5 mm decrease in flare was associated with a 6.3 % increase in the proportion of buds with *Mompha* (Model 4 Fig. 4A). In models where the slopes were allowed to vary between species and site, we saw a significant positive effect of tube length in 6 out of 17 sites with evidence of *Mompha* (Model 6 Fig. 4B) indicating that even within populations, *Mompha* damage was higher on plants with longer floral tubes. In the model where the slope was allowed to vary just by species (excluding site variation), floral flare was negatively associated with *Mompha* in *O. hartwegii* subsp. *pubescens* and *O. lavandulifolia* and tube length was positively associated with increased *Mompha* damage in all species, although the lower bounds of the credible intervals overlapped with zero (Model 5 Fig. 4A). Site to site variation in floral traits and in *Mompha* florivory likely contributed to the large credible intervals in the species-slope model. Estimates for all model parameters are provided in File S2 of Supporting Information.

Pollinator observations largely supported our assumptions of pollinator functional groups (Fig. 3). All hawkmoth-pollinated species (*O. hartwegii* subsp. *pubescens, O. lavandulifolia* and *O. toumeyi*) were visited primarily by the white-lined sphinx moth, *Hyles lineata*, with an average of 1.05 visits per flower per hour. *Manduca quinquemaculata* was observed on both *O. hartwegii* subsp. *pubescens* and *O. lavandulifolia* whereas *Sphinx* sp. was only observed on *O. lavandulifolia*. Except for a few infrequent *Hyles lineata* visits to *O. gayleana* in the morning, hawkmoths were only observed visiting hawkmoth-pollinated flowers (*O. lavandulifolia, O. hartwegii* subsp. *pubescens* and *O. toumeyi*). However, small bees were frequent visitors to flowers of all species.

## Discussion

Our results show striking differences in *Mompha* florivory between sympatric and co-flowering hawkmoth- and bee-pollinated species in *Oenothera* sect. *Calylophus*. Bee-pollinated flowers, which have substantially shorter floral tubes, experience on average 13% less bud *Mompha* florivory than do hawkmoth-pollinated flowers. Further, tube length and floral flare are correlated to *Mompha* florivory within and across species. Even within sites, we see a positive relationship between *Mompha* florivory and floral tube length, suggesting that at local scales, floral traits may be under selection by *Mompha.* The positive association between *Mompha* damage and tube length and the negative association with floral flare suggests that florivory should decouple floral tube length and flare, which are positively correlated floral traits in all five *Oenothera* species. Populations having flowers with shorter tubes and larger flares should decrease the effectiveness of hawkmoth pollination and favour bee-pollination, resulting in a pollinator shift. Thus patterns in *Mompha* florivory indicate that florivore selection can influence the evolution of floral traits at microevolutionary scales with implications for plant-pollinator interactions and speciation at macroevolutionary scales.

There is substantial evidence to show that pollinators influence the evolution of floral traits (Fenster *et al.* 2004 and references therein; Zu *et al.* 2016) and growing evidence that antagonists are also important drivers of floral evolution (Galen 1999; Herrera 2000; Cariveau *et al.*, 2004; Gómez 2008; Carlson and Holsinger 2012; Sun *et al.* 2016). Local adaptation to pollinators can generate floral ecotypes that can eventually segregate as individual species (Kay and Sargent 2009). In the African orchid *Eulophia parviflora*, two morphologically distinct forms are locally adapted to divergent pollinator guilds; long-tongued bees preferentially visit long-spurred forms and beetles primarily visit the short-spurred phenotype (Peter and Johnson 2014). Similarly, in moth-pollinated *Platanthera bifolia*, spur length is geographically correlated with the pollinator fauna (Boberg *et al.* 2013). Here we show that the proportion of buds lost to *Mompha* florivory, and consequently plant fitness, varies with floral morphology. In populations of *O. hartwegii* subsp. *pubescens* and in some populations of *O. lavandulifolia* and *O. toumeyi*, we document a positive association between floral tube length and flower loss to *Mompha* florivory. Geographic variation in floral ecotypes (for e.g. spur length) is often attributed to adaptation to different pollinator communities (Robertson and Wyatt 1990; Anderson *et al.* 2010), but selection based on *Mompha* host-preferences may be equally important in driving floral differentiation.

Herbivore-host preference based on flower size and morphology has been documented in many plants (reviewed in Strauss and Whitall 2006). For example, in *Gelsemium sempervirens* flowers with wider corollas and shorter styles received more floral damage (Leege and Wolfe 2002). Floral size as a determinant of florivory is not surprising considering that bigger flowers offer more resources especially for an internal feeder like *Mompha*, which is restricted to a single flower in its larval stage. Bud feeders predominantly eat reproductive structures (the style and filaments) and rarely damage the petals. In our study, tube length is strongly correlated to style length (R  =  0.99) and flowers with longer tubes likely provide greater nutrition. However, *Mompha* sp. on *Camissoniopsis cheiranthifolia*, showed no apparent preference for larger-flowered plants (Dart and Eckert 2015). Florivores might also discriminate between flowers based on floral shape, as the structure of the flower can limit access to resources and dictate florivore fitness. Floral flare mediates access to nectar for ovipositing *Mompha* adults, however, it is unclear why *Mompha* might prefer flowers with narrow floral flares. Long floral tubes may provide larger feeding enclosures for the larvae. *Mompha* feeding can trigger swelling and ballooning of the floral tubes (Graham 1995; Anstett *et al.* 2014) and these gall-like structures may be easier to induce in longer-tubed flowers. Additionally, factors other than morphology may also contribute to differences in *Mompha* florivory between hawkmoth- and bee-pollinated plants. A shift to morning anthesis can reduce florivory on bee-pollinated plants as *Mompha* adults are crepuscular and putatively lay their eggs in the evening. In wild tobacco, *Nicotiana attenuata*, a shift to morning anthesis mitigates oviposition by *Manduca* moths, which are also active in the evening (Kessler *et al.* 2010). Many evening primroses show inter and intra- specific variation in floral scent (Jogesh *et al.* unpubl. data), which may mediate *Mompha* host choice. Floral volatiles have been shown to function as herbivore attractants and repellents in Texas gourds, *Cucurbita pepo* (Theis and Adler 2012), Canada thistles, *Cirsium arvense* (Theis 2006), and wild parsnips, *Pastinaca sativa* (Jogesh *et al.* 2014). Further, defensive chemistry is a crucial factor in determining host-suitability for many herbivorous insects (Fraenkel 1959; Becerra 1997) and may play an integral role in *Oenothera* sect. *Calylophus*. Complex phenolic compounds including, flavinoids and ellagitannins occur in the leaves, fruits and flowers of multiple *Oenothera* species (Johnson *et al.* 2014). In *O. biennis*, these compounds have been implicated in resistance against generalist and specialist herbivores including the fruit feeding, *Mompha brevivitella* (Johnson *et al.* 2009; Agrawal *et al.* 2012). In addition to constitutive production, herbivore-mediated induction of these complex phenolic defenses results in reduced *Mompha* fruigivory (McArt *et al.* 2013). These phenolic compounds may play an important role in constitutive and induced resistance to *Mompha* florivory and may even be correlated to floral traits in *Oenothera* sect. *Calylophus* but this remains to be evaluated.

**Figure 5:**
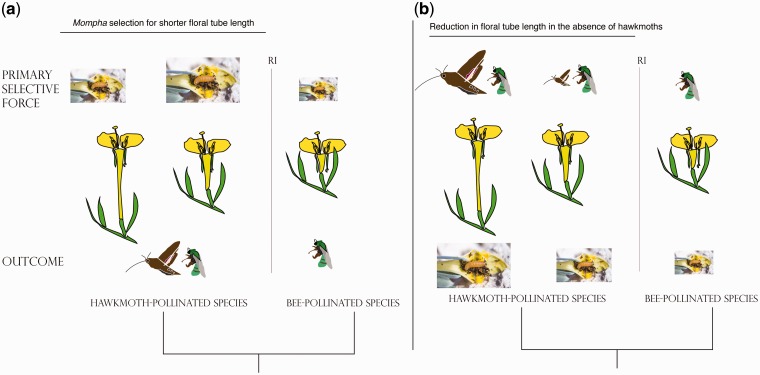
Ecological mechanisms facilitating shifts from hawkmoth to bee pollination. (a) Disruptive selection by *Mompha* herbivory results in a reduction in floral tube length, decreasing the efficacy of hawkmoth pollination. Reproductive isolation (RI) between hawkmoth and bee pollinated plants in sympatry or allopatry is associated with the shift from hawkmoth to bee pollination. (b) The cost of producing larger hawkmoth-pollinated flowers and the spatial variability of hawkmoth pollination, selects for flowers with reduced nectar and hypanthium length resulting a shift from hawkmoth to bee pollination with a secondary loss in *Mompha* herbivory.

Putatively small changes in floral traits driven by herbivore host-preferences can have large effects on pollinator attraction and fidelity. For example, in *Mimulus aurantiacus* (Phrymaceae), a single *cis*-regulatory mutation in the anthocyanin pathway contributes to floral color differentiation between two ecotypes that are pollinated by hawkmoths (yellow) and hummingbirds (red) (Streisfeld and Rausher 2009). Spatially variable florivore selection on floral tube length and floral flare can generate phenotypic divergence in these traits with important implication for pollinator effectiveness (Fig. 5A). Floral morphology in populations with strong *Mompha* herbivore pressure should shift towards shorter floral tubes and wider floral flares. The decoupling of these traits should result in a trait-mismatch between flowers and hawkmoths, favouring phenotypes with shorter tubes and larger floral flares that are more effectively pollinated by bees over hawkmoths. That florivores can generate trait-mismatches between plants and pollinators suggests that plant-pollinator coevolutionary relationships can be modified by spatially variable antagonistic interactions, which is one of the main predictions of the geographic mosaic theory of coevolution (Thompson 1999; Thompson 2005). The efficacy of a particular floral visitor depends on its morphology and behavior (pollen placement) relative to the morphology of the flower it visits (Schemske and Horvitz 1984; Muchhala *et al.* 2007). For example, hawkmoths are highly effective pollinators of the long-tubed *Clarkia brewerii* but are ineffective at pollinating a shorter-tubed sister species, *Clarkia concinna* (Miller *et al.* 2013). In *Oenothera* Sect. *Calylophus*, a shift in phenotype to shorter-tubed flowers may reduce the effectiveness of hawkmoth pollination. Further, a reduction in nectar in shorter-tubed flowers may decrease the attractiveness of flowers to hawkmoths. Subsequent pollinator-driven selection (e.g. on the timing of anthesis) may reinforce floral differentiation resulting in flowers that are either predominantly pollinated by hawkmoths or by bees.

Alternatively, adaptation to differential pollinator communities in allopatric populations may have facilitated pollinator shifts with a subsequent reduction in *Mompha* florivory (Fig. 5B). Pollinator-mediated selection is the most frequently cited driver of floral differentiation and consecutive pollinator shifts (Van der Niet and Johnson 2012). In many species, geographic variation in floral traits is associated with distinct pollinator communities (Johnson 2006; Anderson and Johnson 2008, 2009; Pauw *et al.*, 2009; Peter and Johnson, 2014). For example, in *Calceolaria polyrhiza*, floral traits vary across the range of the species, based on the mechanical fit between the flowers and the main pollinator (Cosacov *et al.* 2013). In *Oenothera* Sect. *Calylophus*, co-occurring hawkmoth- and bee-pollinated species currently share the same pollinator community and are yet strongly differentiated in morphology. While morphology is predictive of hawkmoth visitations in this group (Fig. 2), it is interesting to note that flowers remain open for 36-60 hours and we observed bee visits on all focal species. *Oenothera* Sect. *Calylophus* exhibit traits associated with the classic hawkmoth-syndrome (larger corollas, tube lengths, nectar and scent production) but can remain open for multiple days and display UV reflectance patterns (Towner 1977) and therefore are apparent to bee visitors. For hawkmoth-pollinated members of *Oenothera* sect. *Calylophus*, bees may provide ancillary pollination services in years when hawkmoths are scarce (see Barthell and Knops 1997). Hawkmoth pollination increases outcrossing rates (Herrera 1987; Brunet and Sweet 2006; Brunet and Holmquist 2009) and can substantially increase plant fitness (Rhodes *et al.*, in review) but large hawkmoth-flowers and high nectar volumes can be costly to produce, especially in the resource-poor Chihuahuan Desert where the majority of these taxa occur. In populations that were historically allopatric, the cost of hawkmoth pollination and the spatial and temporal patchiness of hawkmoth abundance (Miller 1981; Campbell *et al.* 1997; Artz *et al.* 2010) may have driven shifts from hawkmoth to bee pollination accompanied by a reduction in *Mompha* florivory.

At the centre of its diversity, hawkmoth- and bee-pollinated *Oenothera* Sect. *Calylophus* are broadly sympatric with species often co-flowering in close proximity (Clinebell *et al.* 2004). However, current distributions are not necessarily representative of past ranges, which have likely undergone expansions and contractions owing to historic fluctuations in temperature and precipitation. Secondary range expansion after allopatric speciation may explain the current geographic distributions. Given that hawkmoth pollination is ancestral to sect. *Calylophus* and that there are only two shifts from hawkmoth to bee pollination, the primary mechanism of diversification in this group is likely allopatric. However, the independent shifts to bee pollination, one in the ancestor of subsect. *Calylophus* and the other in the ancestor(s) of *O. tubicula* (Cooper 2016), may have occurred in allopatry or sympatry. Nonetheless, substantial differentiation in floral morphology between hawkmoth- and bee-pollinated species suggests that biotic interactions have played a crucial role in the evolution of these traits and taxa. The remarkable difference in *Mompha* florivory between co-flowering and sympatric hawkmoth- and bee-pollinated species suggests that shifts to bee pollination are accompanied by a substantial reduction in florivory. Further, the strong relationship between florivory and morphology indicates that florivores may have played a key role in floral differentiation and in subsequent pollinator shifts.

## Conclusions

In conclusion, we show that floral traits implicated in pollinator attraction predict florivore damage within sites, species and even across a group of closely related species. We are just beginning to understand how conflicts in herbivore- and pollinator-mediated selection drive trait evolution at a microevolutionary scale, and few studies examine how natural selection translates to larger macroevolutionary patterns of trait evolution. By documenting disruptive selection within species and trait differentiation among species, we can document the mechanisms by which species interactions can influence diversification (Althoff *et al.* 2014). While we focus on a single section of evening primroses in this study, the evolutionary transition from xenogamy to autogamy is recurrent across Onagraceae (Raven 1979; Cruden and Lyon 1989; Button *et al.* 2012). *Mompha*, which are ubiquitous on Onagraceae, may have been an important selective force in driving the switch from larger, insect-pollinated flowers to smaller, autogamous flowers in this group. Future studies that combine robust experimental approaches with phylogenetic comparisons can help elucidate the role of antagonistic and mutualistic interactions in the diversification of floral traits and mating systems.

## Sources of Funding 

Funding was provided by the National Science Foundation (DEB 1342873, DEB 1342792, to K.A.S. and R.A.R), and the Chicago Botanic Garden Division of Plant Science and Conservation.

## Contributions by the Authors

R.A.R. and K.A.S. initiated the study. T.J., R.P.O. and K.A.S. collected the data. T.J. analysed the data and wrote the manuscript. All authors contributed to discussion and revisions on the manuscript.

## Conflicts of Interest Statement

No conflicts of interest.

## Supplementary Material

Supplementary Data
