# Supplementary material for: Herbivory as an important selective force in the evolution of floral traits and pollinator shifts
Source: AoB Plants. 2016 Dec 22;9(1):plw088. doi: 10.1093/aobpla/plw088 (PMC5499749; doi:10.1093/aobpla/plw088)
Supplement: Supplementary Data [file plw088_Supp.zip › plw088_Supp/Supporting information File 2.docx]

Posterior coefficient estimates from hierarchical analysis for models specified in Table 2. Estimates are indicated by the median and the 95% credible interval (CI). Estimates whose 95% equal-tailed credible interval does not include zero are indicated in boldface type. The potential scale reduction factor (R.hat) is also provided for all estimated coefficients.

|  | Intercept/Slope | Median Posterior probability | Lower CI 2.5 | Upper CI 97.5 | R-hat |
| --- | --- | --- | --- | --- | --- |
| Model 1 | Section: *Calylophus* | 0.12 | -153.83 | 154.77 | 1.00 |
|  | Section: *Salpingia* +*O. toumeyi* | 0.10 | -0.07 | 0.27 | 1.00 |
|  | Species: *O. lavandulifolia* | **0.10** | **0.07** | **0.12** | 1.00 |
|  | Species: *O. tubicula tubicula* | 0.02 | -0.01 | 0.05 | 1.00 |
|  | Species: *O. harytwegii pubescens* | **0.27** | **0.24** | **0.30** | 1.00 |
|  | Species: *O. gayleana* | 0.03 | 0.00 | 0.05 | 1.00 |
|  | Species: *O. toumeyi* | **0.09** | **0.04** | **0.13** | 1.00 |
| Variance | | 0.16 | 0.15 | 0.17 | 1.00 |
| Model2 | Section: *Calylophus* | 0.01 | -166.57 | 166.72 | 1.00 |
|  | Section: *Salpingia* +*O. toumeyi* | 0.11 | -99.70 | 100.86 | 1.00 |
|  | Site: *O. tubicula* -- PineSprTX | 0.01 | -0.04 | 0.07 | 1.00 |
|  | Site: *O. lavandulifolia* -- SAlpine | **0.33** | **0.28** | **0.38** | 1.00 |
|  | Site: *O. lavandulifolia* -- SlickRock | **0.06** | **0.01** | **0.11** | 1.00 |
|  | Site: *O. lavandulifolia* -- TanSeeps UT | 0.01 | -0.04 | 0.06 | 1.00 |
|  | Site: *O. lavandulifolia* -- SilverCreek | 0.01 | -0.06 | 0.07 | 1.00 |
|  | Site: *O. lavandulifolia* -- DCW | **0.07** | **0.03** | **0.11** | 1.00 |
|  | Site: *O. tubicula* --NinePtMesa | 0.06 | -0.02 | 0.14 | 1.00 |
|  | Site: *O. tubicula* --BlackRvVillage | 0.05 | -0.02 | 0.11 | 1.00 |
|  | Site: *O. tubicula* --BoxCanyonRd | 0.01 | -0.04 | 0.06 | 1.00 |
|  | Site: *O. tubicula* --Picacho, NM | 0.01 | -0.06 | 0.07 | 1.00 |
|  | Site: *O. harytwegii pubescens* -- SStockton | **0.33** | **0.28** | **0.39** | 1.00 |
|  | Site: *O. harytwegii pubescens* -- SierraDiablo | **0.23** | **0.17** | **0.28** | 1.00 |
|  | Site: *O. harytwegii pubescens* -- Hwy82 | **0.29** | **0.21** | **0.37** | 1.00 |
|  | Site: *O. harytwegii pubescens* -- Taiban | **0.24** | **0.19** | **0.28** | 1.00 |
|  | Site: *O. gayleana* -- GaslineRd | 0.02 | -0.04 | 0.09 | 1.00 |
|  | Site: *O. gayleana* -- SevenRivers | 0.04 | -0.01 | 0.09 | 1.00 |
|  | Site: *O. gayleana* -- TrigRanch, NM | 0.01 | -0.04 | 0.06 | 1.00 |
|  | Site: *O. gayleana* -- CrotonCamp | 0.05 | -0.01 | 0.10 | 1.00 |
|  | Site: *O. toumeyi* -- PinaryCanyonAZ | 0.04 | -0.01 | 0.09 | 1.00 |
|  | Site: *O. toumeyi* -- CarrCanyonAZ | **0.14** | **0.08** | **0.19** | 1.00 |
|  | Species: *O.*  *lavandulifolia* | 0.10 | 0.04 | 0.15 | 1.00 |
|  | Species: *O.*  *tubicula* *tubicula* | 0.14 | -156.48 | 158.21 | 1.00 |
|  | Species: *O.*  *harytwegii pubescens* | 0.12 | -156.13 | 158.05 | 1.00 |
|  | Species: *O.*  *gayleana* | 0.08 | -157.23 | 157.23 | 1.00 |
|  | Species: *O.*  *toumeyi* | 0.13 | -157.31 | 157.57 | 1.00 |
| Variance | | 0.14 | 0.13 | 0.15 | 1.00 |
| Model 3 | Section: *Calylophus* | 0.01 | -0.02 | 0.03 | 1.00 |
|  | Section: *Salpingia* +*O. toumeyi* | **0.04** | **0.01** | **0.07** | 1.00 |
|  | syndrome | **0.12** | **0.09** | **0.15** | 1.00 |
| Variance | | 0.17 | 0.16 | 0.18 | 1.00 |
| Model 4 | Section: *Calylophus* | **0.07** | **0.04** | **0.10** | 1.00 |
|  | Section: *Salpingia* +*O. toumeyi* | **0.12** | **0.09** | **0.14** | 1.00 |
|  | corolla | 0.02 | -0.02 | 0.06 | 1.00 |
|  | flare | **-0.06** | **-0.11** | **-0.02** | 1.00 |
|  | herk | -0.02 | -0.04 | 0.01 | 1.00 |
|  | nectar | -0.03 | -0.06 | -0.01 | 1.00 |
|  | tube | **0.13** | **0.09** | **0.17** | 1.00 |
| Variance | | 0.16 | 0.15 | 0.18 | 1.00 |
| Model 5 | corolla | 0.00 | -0.04 | 0.05 | 1.06 |
|  | flare | -0.04 | -0.10 | 0.03 | 1.07 |
|  | tube | -0.01 | -0.06 | 0.07 | 1.02 |
|  | herk | 0.00 | -0.04 | 0.02 | 1.01 |
|  | nectar | 0.00 | -0.03 | 0.03 | 1.03 |
|  | Species: *O.*  *gayleana* *corolla | 0.00 | -0.06 | 0.05 | 1.03 |
|  | Species: *O.*  *gayleana* *flare | -0.02 | -0.08 | 0.08 | 1.06 |
|  | Species: *O.*  *gayleana* *tube | 0.00 | -0.04 | 0.02 | 1.00 |
|  | Species: *O.*  *gayleana* *herk | 0.00 | -0.06 | 0.05 | 1.03 |
|  | Species: *O.*  *gayleana* *nectar | 0.03 | -0.05 | 0.11 | 1.01 |
|  | Species: *O.*  *gayleana* | 0.03 | -0.07 | 0.15 | 1.02 |
|  | Species: *O.*  *harytwegii pubescens**corolla | 0.00 | -0.04 | 0.06 | 1.06 |
|  | Species: *O.*  *harytwegii pubescens**flare | **-0.05** | **-0.11** | **0.00** | 1.03 |
|  | Species: *O.*  *harytwegii pubescens**herk | 0.00 | -0.03 | 0.04 | 1.09 |
|  | Species: *O.*  *harytwegii pubescens**nectar | 0.00 | -0.04 | 0.02 | 1.05 |
|  | Species: *O.*  *harytwegii pubescens**tube | 0.02 | -0.05 | 0.09 | 1.07 |
|  | Species: *O.*  *harytwegii pubescens* | **0.30** | **0.24** | **0.36** | 1.20 |
|  | Species: *O.*  *lavandulifolia* *corolla | 0.00 | -0.04 | 0.05 | 1.01 |
|  | Species: *O.*  *lavandulifolia* *flare | **-0.07** | **-0.12** | **-0.02** | 1.31 |
|  | Species: *O.*  *lavandulifolia* *herk | 0.00 | -0.03 | 0.02 | 1.03 |
|  | Species: *O.*  *lavandulifolia* *nectar | 0.00 | -0.03 | 0.02 | 1.03 |
|  | Species: *O.*  *lavandulifolia* *tube | 0.02 | -0.04 | 0.09 | 1.01 |
|  | Species: *O.*  *lavandulifolia* | **0.16** | **0.08** | **0.23** | 1.03 |
|  | Species: *O.*  *toumeyi* *corolla | 0.00 | -0.05 | 0.05 | 1.01 |
|  | Species: *O.*  *toumeyi* *flare | -0.05 | -0.12 | 0.01 | 1.02 |
|  | Species: *O.*  *toumeyi* *herk | 0.00 | -0.05 | 0.02 | 1.02 |
|  | Species: *O.*  *toumeyi* *nectar | -0.01 | -0.05 | 0.02 | 1.01 |
|  | Species: *O.*  *toumeyi* *tube | 0.03 | -0.04 | 0.10 | 1.02 |
|  | Species: *O.*  *toumeyi* | **0.10** | **0.03** | **0.18** | 1.01 |
|  | Species: *O.*  *tubicula* *tubicula**corolla | 0.00 | -0.05 | 0.06 | 1.00 |
|  | Species: *O.*  *tubicula* *tubicula**flare | -0.02 | -0.09 | 0.09 | 1.03 |
|  | Species: *O.*  *tubicula* *tubicula**herk | 0.00 | -0.05 | 0.02 | 1.04 |
|  | Species: *O.*  *tubicula* *tubicula**nectar | 0.00 | -0.06 | 0.05 | 1.02 |
|  | Species: *O.*  *tubicula* *tubicula**tube | 0.03 | -0.03 | 0.12 | 1.04 |
|  | Species: *O.*  *tubicula* *tubicula* | 0.02 | -0.07 | 0.15 | 1.01 |
| Variance | | 0.15 | 0.14 | 0.16 | 1.00 |
| Model 6 | corolla | -0.01 | -0.06 | 0.03 | 1.32 |
|  | flare | 0.00 | -0.03 | 0.05 | 1.33 |
|  | tube | **0.07** | **0.01** | **0.13** | 1.18 |
|  | herk | 0.00 | -0.03 | 0.03 | 1.15 |
|  | nectar | -0.01 | -0.04 | 0.01 | 1.05 |
|  | Site: *O. gayleana* -- CrotonCamp*corolla | -0.02 | -0.07 | 0.02 | 1.02 |
|  | Site: *O. gayleana* -- CrotonCamp*flare | 0.02 | -0.02 | 0.12 | 1.28 |
|  | Site: *O. gayleana* -- CrotonCamp*herk | 0.00 | -0.03 | 0.03 | 1.11 |
|  | Site: *O. gayleana* -- CrotonCamp*nectar | -0.01 | -0.05 | 0.02 | 1.11 |
|  | Site: *O. gayleana* -- CrotonCamp*tube | 0.06 | -0.08 | 0.19 | 1.08 |
|  | Site: *O. gayleana* -- CrotonCamp | 0.11 | -0.03 | 0.25 | 1.01 |
|  | Site: *O. gayleana* -- GaslineRd*corolla | -0.02 | -0.07 | 0.03 | 1.01 |
|  | Site: *O. gayleana* -- GaslineRd*flare | 0.01 | -0.05 | 0.08 | 1.02 |
|  | Site: *O. gayleana* -- GaslineRd*herk | 0.00 | -0.02 | 0.03 | 1.01 |
|  | Site: *O. gayleana* -- GaslineRd*nectar | -0.01 | -0.05 | 0.03 | 1.16 |
|  | Site: *O. gayleana* -- GaslineRd*tube | 0.06 | -0.07 | 0.20 | 1.08 |
|  | Site: *O. gayleana* -- GaslineRd | 0.08 | -0.06 | 0.22 | 1.37 |
|  | Site: *O. gayleana* -- SevenRivers*corolla | -0.02 | -0.07 | 0.03 | 1.10 |
|  | Site: *O. gayleana* -- SevenRivers*flare | 0.01 | -0.05 | 0.06 | 1.02 |
|  | Site: *O. gayleana* -- SevenRivers*herk | 0.00 | -0.03 | 0.03 | 1.03 |
|  | Site: *O. gayleana* -- SevenRivers*nectar | -0.01 | -0.05 | 0.03 | 1.04 |
|  | Site: *O. gayleana* -- SevenRivers*tube | 0.05 | -0.07 | 0.18 | 1.02 |
|  | Site: *O. gayleana* -- SevenRivers | 0.09 | -0.05 | 0.21 | 1.10 |
|  | Site: *O. gayleana* -- TrigRanch, NM*corolla | -0.02 | -0.07 | 0.03 | 1.03 |
|  | Site: *O. gayleana* -- TrigRanch, NM*flare | 0.01 | -0.04 | 0.09 | 1.15 |
|  | Site: *O. gayleana* -- TrigRanch, NM*herk | 0.00 | -0.02 | 0.03 | 1.14 |
|  | Site: *O. gayleana* -- TrigRanch, NM*nectar | 0.00 | -0.04 | 0.03 | 1.19 |
|  | Site: *O. gayleana* -- TrigRanch, NM*tube | 0.05 | -0.07 | 0.20 | 1.14 |
|  | Site: *O. gayleana* -- TrigRanch, NM | 0.06 | -0.06 | 0.20 | 1.20 |
|  | Site: *O. harytwegii pubescens* -- Hwy82*corolla | -0.02 | -0.07 | 0.02 | 1.16 |
|  | Site: *O. harytwegii pubescens* -- Hwy82*flare | 0.01 | -0.05 | 0.09 | 1.16 |
|  | Site: *O. harytwegii pubescens* -- Hwy82*herk | 0.01 | -0.02 | 0.04 | 1.15 |
|  | Site: *O. harytwegii pubescens* -- Hwy82*nectar | -0.01 | -0.07 | 0.01 | 1.18 |
|  | Site: *O. harytwegii pubescens* -- Hwy82*tube | **0.11** | **0.01** | **0.22** | 1.24 |
|  | Site: *O. harytwegii pubescens* -- Hwy82 | **0.20** | **0.13** | **0.32** | 1.10 |
|  | Site: *O. harytwegii pubescens* -- SierraDiablo*corolla | -0.02 | -0.06 | 0.03 | 1.24 |
|  | Site: *O. harytwegii pubescens* -- SierraDiablo*flare | 0.01 | -0.04 | 0.07 | 1.13 |
|  | Site: *O. harytwegii pubescens* -- SierraDiablo*herk | 0.00 | -0.02 | 0.03 | 1.17 |
|  | Site: *O. harytwegii pubescens* -- SierraDiablo*nectar | -0.01 | -0.04 | 0.02 | 1.19 |
|  | Site: *O. harytwegii pubescens* -- SierraDiablo*tube | **0.08** | **0.00** | **0.15** | 1.16 |
|  | Site: *O. harytwegii pubescens* -- SierraDiablo | **0.15** | **0.09** | **0.24** | 1.21 |
|  | Site: *O. harytwegii pubescens* -- SStockton*corolla | -0.02 | -0.09 | 0.01 | 1.16 |
|  | Site: *O. harytwegii pubescens* -- SStockton*flare | 0.00 | -0.08 | 0.06 | 1.26 |
|  | Site: *O. harytwegii pubescens* -- SStockton*herk | 0.01 | -0.02 | 0.04 | 1.18 |
|  | Site: *O. harytwegii pubescens* -- SStockton*nectar | -0.01 | -0.07 | 0.01 | 1.19 |
|  | Site: *O. harytwegii pubescens* -- SStockton*tube | **0.13** | **0.01** | **0.25** | 1.17 |
|  | Site: *O. harytwegii pubescens* -- SStockton | **0.26** | **0.19** | **0.36** | 1.23 |
|  | Site: *O. harytwegii pubescens* -- Taiban*corolla | -0.02 | -0.08 | 0.01 | 1.19 |
|  | Site: *O. harytwegii pubescens* -- Taiban*flare | 0.00 | -0.13 | 0.06 | 1.17 |
|  | Site: *O. harytwegii pubescens* -- Taiban*herk | 0.01 | -0.02 | 0.04 | 1.18 |
|  | Site: *O. harytwegii pubescens* -- Taiban*nectar | -0.01 | -0.07 | 0.01 | 1.17 |
|  | Site: *O. harytwegii pubescens* -- Taiban*tube | **0.11** | **0.01** | **0.21** | 1.18 |
|  | Site: *O. harytwegii pubescens* -- Taiban | **0.20** | **0.13** | **0.29** | 1.17 |
|  | Site: *O. lavandulifolia* -- SAlpine*corolla | -0.02 | -0.08 | 0.02 | 1.21 |
|  | Site: *O. lavandulifolia* -- SAlpine*flare | 0.02 | -0.04 | 0.14 | 1.21 |
|  | Site: *O. lavandulifolia* -- SAlpine*herk | 0.01 | -0.02 | 0.04 | 1.18 |
|  | Site: *O. lavandulifolia* -- SAlpine*nectar | -0.01 | -0.05 | 0.01 | 1.17 |
|  | Site: *O. lavandulifolia* -- SAlpine*tube | **0.14** | **0.01** | **0.24** | 1.19 |
|  | Site: *O. lavandulifolia* -- SAlpine | **0.23** | **0.16** | **0.33** | 1.18 |
|  | Site: *O. lavandulifolia* -- SilverCreek*corolla | -0.02 | -0.06 | 0.04 | 1.21 |
|  | Site: *O. lavandulifolia* -- SilverCreek*flare | 0.01 | -0.05 | 0.07 | 1.15 |
|  | Site: *O. lavandulifolia* -- SilverCreek*herk | 0.00 | -0.03 | 0.02 | 1.18 |
|  | Site: *O. lavandulifolia* -- SilverCreek*nectar | 0.00 | -0.04 | 0.03 | 1.08 |
|  | Site: *O. lavandulifolia* -- SilverCreek*tube | 0.01 | -0.07 | 0.09 | 1.61 |
|  | Site: *O. lavandulifolia* -- SilverCreek | 0.01 | -0.08 | 0.08 | 1.18 |
|  | Site: *O. lavandulifolia* -- SlickRock*corolla | -0.02 | -0.06 | 0.04 | 1.11 |
|  | Site: *O. lavandulifolia* -- SlickRock*flare | 0.02 | -0.02 | 0.09 | 1.15 |
|  | Site: *O. lavandulifolia* -- SlickRock*herk | 0.00 | -0.02 | 0.03 | 1.08 |
|  | Site: *O. lavandulifolia* -- SlickRock*nectar | 0.00 | -0.03 | 0.03 | 1.06 |
|  | Site: *O. lavandulifolia* -- SlickRock*tube | 0.03 | -0.05 | 0.09 | 1.09 |
|  | Site: *O. lavandulifolia* -- SlickRock | 0.03 | -0.05 | 0.10 | 1.12 |
|  | Site: *O. lavandulifolia* -- TanSeeps UT*corolla | -0.02 | -0.06 | 0.04 | 1.36 |
|  | Site: *O. lavandulifolia* -- TanSeeps UT*flare | 0.01 | -0.04 | 0.07 | 1.27 |
|  | Site: *O. lavandulifolia* -- TanSeeps UT*herk | 0.00 | -0.03 | 0.02 | 1.51 |
|  | Site: *O. lavandulifolia* -- TanSeeps UT*nectar | 0.00 | -0.03 | 0.03 | 1.43 |
|  | Site: *O. lavandulifolia* -- TanSeeps UT*tube | 0.02 | -0.08 | 0.10 | 1.08 |
|  | Site: *O. lavandulifolia* -- TanSeeps UT | 0.01 | -0.07 | 0.08 | 1.06 |
|  | Site: *O. toumeyi* -- CarrCanyonAZ*corolla | -0.02 | -0.07 | 0.02 | 1.09 |
|  | Site: *O. toumeyi* -- CarrCanyonAZ*flare | 0.01 | -0.06 | 0.06 | 1.09 |
|  | Site: *O. toumeyi* -- CarrCanyonAZ*herk | 0.00 | -0.03 | 0.02 | 1.12 |
|  | Site: *O. toumeyi* -- CarrCanyonAZ*nectar | -0.01 | -0.06 | 0.01 | 1.12 |
|  | Site: *O. toumeyi* -- CarrCanyonAZ*tube | 0.06 | 0.00 | 0.14 | 1.09 |
|  | Site: *O. toumeyi* -- CarrCanyonAZ | **0.09** | **0.03** | **0.16** | 1.10 |
|  | Site: *O. toumeyi* -- PinaryCanyonAZ*corolla | -0.02 | -0.06 | 0.04 | 1.07 |
|  | Site: *O. toumeyi* -- PinaryCanyonAZ*flare | 0.01 | -0.06 | 0.06 | 1.09 |
|  | Site: *O. toumeyi* -- PinaryCanyonAZ*herk | 0.00 | -0.03 | 0.02 | 1.08 |
|  | Site: *O. toumeyi* -- PinaryCanyonAZ*nectar | 0.00 | -0.04 | 0.03 | 1.10 |
|  | Site: *O. toumeyi* -- PinaryCanyonAZ*tube | 0.02 | -0.06 | 0.09 | 1.10 |
|  | Site: *O. toumeyi* -- PinaryCanyonAZ | 0.03 | -0.04 | 0.09 | 1.08 |
|  | Site: *O. tubicula* -- PineSprTX*corolla | -0.02 | -0.07 | 0.03 | 1.10 |
|  | Site: *O. tubicula* -- PineSprTX*flare | 0.01 | -0.04 | 0.09 | 1.12 |
|  | Site: *O. tubicula* -- PineSprTX*herk | 0.00 | -0.02 | 0.03 | 1.09 |
|  | Site: *O. tubicula* -- PineSprTX*nectar | 0.00 | -0.05 | 0.03 | 1.09 |
|  | Site: *O. tubicula* -- PineSprTX*tube | 0.05 | -0.07 | 0.20 | 1.07 |
|  | Site: *O. tubicula* -- PineSprTX | 0.07 | -0.05 | 0.21 | 1.08 |
|  | Site: *O. tubicula* --BlackRvVillage*corolla | -0.02 | -0.07 | 0.03 | 1.10 |
|  | Site: *O. tubicula* --BlackRvVillage*flare | 0.01 | -0.04 | 0.09 | 1.09 |
|  | Site: *O. tubicula* --BlackRvVillage*herk | 0.00 | -0.02 | 0.03 | 1.12 |
|  | Site: *O. tubicula* --BlackRvVillage*nectar | -0.01 | -0.05 | 0.02 | 1.07 |
|  | Site: *O. tubicula* --BlackRvVillage*tube | 0.06 | -0.06 | 0.19 | 1.09 |
|  | Site: *O. tubicula* --BlackRvVillage | 0.09 | -0.03 | 0.21 | 1.04 |
|  | Site: *O. tubicula* --BoxCanyonRd*corolla | -0.02 | -0.06 | 0.03 | 1.03 |
|  | Site: *O. tubicula* --BoxCanyonRd*flare | 0.01 | -0.04 | 0.08 | 1.05 |
|  | Site: *O. tubicula* --BoxCanyonRd*herk | 0.00 | -0.03 | 0.03 | 1.06 |
|  | Site: *O. tubicula* --BoxCanyonRd*nectar | 0.00 | -0.04 | 0.03 | 1.06 |
|  | Site: *O. tubicula* --BoxCanyonRd*tube | 0.05 | -0.08 | 0.17 | 1.03 |
|  | Site: *O. tubicula* --BoxCanyonRd | 0.05 | -0.06 | 0.17 | 1.04 |
|  | Site: *O. tubicula* --NinePtMesa*corolla | -0.02 | -0.07 | 0.02 | 1.03 |
|  | Site: *O. tubicula* --NinePtMesa*flare | 0.01 | -0.04 | 0.10 | 1.03 |
|  | Site: *O. tubicula* --NinePtMesa*herk | 0.00 | -0.02 | 0.03 | 1.01 |
|  | Site: *O. tubicula* --NinePtMesa*nectar | -0.01 | -0.05 | 0.02 | 1.04 |
|  | Site: *O. tubicula* --NinePtMesa*tube | 0.07 | -0.04 | 0.22 | 1.02 |
|  | Site: *O. tubicula* --NinePtMesa | 0.12 | -0.01 | 0.26 | 1.02 |
|  | Site: *O. tubicula* --Picacho, NM*corolla | -0.02 | -0.07 | 0.03 | 1.03 |
|  | Site: *O. tubicula* --Picacho, NM*flare | 0.01 | -0.04 | 0.09 | 1.03 |
|  | Site: *O. tubicula* --Picacho, NM*herk | 0.00 | -0.03 | 0.03 | 1.04 |
|  | Site: *O. tubicula* --Picacho, NM*nectar | -0.01 | -0.05 | 0.03 | 1.03 |
|  | Site: *O. tubicula* --Picacho, NM*tube | 0.06 | -0.07 | 0.20 | 1.05 |
|  | Site: *O. tubicula* --Picacho, NM | 0.07 | -0.07 | 0.22 | 1.02 |
| Variance | | 0.13 | 0.12 | 0.14 | 1.00 |
