# Supplementary material for: Herbivory as an important selective force in the evolution of floral traits and pollinator shifts
Source: AoB Plants. 2016 Dec 22;9(1):plw088. doi: 10.1093/aobpla/plw088 (PMC5499749; doi:10.1093/aobpla/plw088)
Supplement: Supplementary Data [file plw088_Supp.zip › plw088_Supp/Supporting information File 1.docx]

**# Variables used in models:**

# y : Proportion of buds with Mompha

# section: (1) Calylophus (2) Salpingia + *O. toumeyi*

# species: (1) O. lavandulifolia (2) O. tubicula tubicula (3) O. harytwegii pubescens (4) O. gayleana (5) O. toumeyi

# site: (1) O. tubicula -- PineSprTX (2) O. lavandulifolia -- SAlpine (3) O. lavandulifolia -- SlickRock (4) O. lavandulifolia – TanSeeps UT (5) O. lavandulifolia -- SilverCreek (6) O. lavandulifolia -- DCW (6) O. tubicula --NinePtMesa (7) O. tubicula --BlackRvVillage (8) O. tubicula --BoxCanyonRd (9) O. tubicula --Picacho, NM (10) O. harytwegii pubescens -- SStockton (11) O. harytwegii pubescens -- SierraDiablo (12) O. harytwegii pubescens -- Hwy82 (13) O. harytwegii pubescens -- Taiban (14) O. gayleana -- GaslineRd (15) O. gayleana -- SevenRivers (16) O. gayleana -- TrigRanch, NM (17) O. gayleana -- CrotonCamp (18) O. toumeyi -- PinaryCanyonAZ (19) O. toumeyi -- CarrCanyonAZ

syndrome: bee – 0 , hawkmoth – 1

# n.samp = 410

# n.section =2

# n.species = 5

# n.sites =19

# corolla: standardized diameter of the corolla

# flare: standardized opening of the floral flare

# tube: standardized length of the floral flare

# herk: standardized separation between stigma and anthers

# nectar: standardized volume of nectar

**# MODEL 1**

################## JAGS MODEL #1

data.jags<-list('n.samp' = n.samp ,'y'= mompha,

'species' =species,

'section' = section,

'n.species' = n.species,

'n.section' = n.section)

data.inits <- function (){

list (beta.section=rnorm(n.section),

beta.species=rnorm(n.species),

sigma.y=runif(1))

}

data.parameters <- c ("beta.section","beta.species",

"sigma.y")

data.jags.model <- jags.model(file ='/Users/taniajogesh/Dropbox/CBG Dimensions/Field Data/R files/CalyModel1.bug',

data = data.jags,

inits = data.inits,

n.chains = 4, n.adapt = 1000)

sdata.jags.model <- coda.samples(data.jags.model, data.parameters, n.iter = 100000)

# Posterior medians and CIs

round(summary(window(sdata.jags.model, start = 5000))$quantiles[,c(3, 1, 5)], 3)

# Gelman diagnostics

gelman.diag(sdata.jags.model)$psrf

#=======================================

**model {**

**for (i in 1:n.samp){**

**y[i] ~ dnorm(y.hat[i], tau.y)**

**y.hat[i] <- beta.species[species[i]]**

**}**

**for (k in 1:n.species) {**

**beta.species[k]~dnorm(mu.species[k], tau.species)**

**mu.species[k]<-beta.section[section[k]]**

**}**

**for (j in 1:n.section) {**

**beta.section[j]~dnorm(mu.section, tau.section)**

**}**

**mu.section~ dnorm(0, 0.0001)**

**tau.section <- pow(sigma.section, -2)**

**sigma.section ~ dunif (0, 100)**

**tau.species <- pow(sigma.species, -2)**

**sigma.species ~ dunif (0, 100)**

**tau.y<- pow(sigma.y, -2)**

**sigma.y ~ dunif (0, 100)**

**}**

#=======================================

**# MODEL 2**

################## JAGS MODEL #2

data.jags<-list('n.samp' = n.samp ,'y'= mompha,

'species' =species,

'section' = section,

'site' = site,

'n.species' = n.species,

'n.site' = n.site,

'n.section' = n.section)

data.inits <- function (){

list (beta.section=rnorm(n.section),

beta.species=rnorm(n.species),

beta.site=rnorm(n.site),

sigma.y=runif(1))

}

data.parameters <- c ("beta.section","beta.species","beta.site",

"sigma.y")

data.jags.model <- jags.model(file ='/Users/taniajogesh/Dropbox/CBG Dimensions/Field Data/R files/CalyModel2.bug',

data = data.jags,

inits = data.inits,

n.chains = 4, n.adapt = 1000)

sdata.jags.model <- coda.samples(data.jags.model, data.parameters, n.iter = 100000)

round(summary(window(sdata.jags.model, start = 5000))$quantiles[,c(3, 1, 5)], 3)

gelman.diag(sdata.jags.model)$psrf

#==========================================

**model {**

**for (i in 1:n.samp){**

**y[i] ~ dnorm(y.hat[i], tau.y)**

**y.hat[i] <- beta.site[site[i]]**

**}**

**for (l in 1:n.site) {**

**beta.site[l]~dnorm(mu.site[l], tau.site)**

**mu.site[l]<-beta.species[species[l]]**

**}**

**for (k in 1:n.species) {**

**beta.species[k]~dnorm(mu.species[k], tau.species)**

**mu.species[k]<-beta.section[section[k]]**

**}**

**for (j in 1:n.section) {**

**beta.section[j]~dnorm(mu.section, tau.section)**

**}**

**mu.section~ dnorm(0, 0.0001)**

**tau.section <- pow(sigma.section, -2)**

**sigma.section ~ dunif (0, 100)**

**tau.species <- pow(sigma.species, -2)**

**sigma.species ~ dunif (0, 100)**

**tau.site <- pow(sigma.site, -2)**

**sigma.site ~ dunif (0, 100)**

**tau.y<- pow(sigma.y, -2)**

**sigma.y ~ dunif (0, 100)**

**}**

#==========================================

**# MODEL 3**

################## JAGS MODEL #3

data.jags<-list('n.samp' = n.samp ,'y'= mompha,

'syndrome' =syndrom,

'section' = section,

'n.section' = n.section)

data.inits <- function (){

list (beta.syndrome=rnorm(1),

beta.section=rnorm(n.section),

sigma.y=runif(1))

}

data.parameters <- c ("beta.section","beta.syndrome",

"sigma.y")

data.jags.model <- jags.model(file ='/Users/taniajogesh/Dropbox/CBG Dimensions/Field Data/R files/CalyModel3.bug',

data = data.jags,

inits = data.inits,

n.chains = 4, n.adapt = 1000)

sdata.jags.model <- coda.samples(data.jags.model, data.parameters, n.iter = 100000)

round(summary(window(sdata.jags.model, start = 5000))$quantiles[,c(3, 1, 5)], 3)

gelman.diag(sdata.jags.model)$psrf

#==========================================

**model {**

**for (i in 1:n.samp){**

**y[i] ~ dnorm(y.hat[i], tau.y)**

**y.hat[i] <- beta.section[section[i]] + beta.syndrome*syndrome[i]**

**}**

**beta.syndrome~dnorm(0, 0.0001)**

**tau.y <- pow(sigma.y, -2)**

**sigma.y ~ dunif (0, 100)**

**for (j in 1:n.section) {**

**beta.section[j]~dnorm(mu.section, tau.section)**

**}**

**mu.section~ dnorm(0, 0.0001)**

**tau.section <- pow(sigma.section, -2)**

**sigma.section ~ dunif (0, 100)**

**}**

#==========================================

**# MODEL 4**

################## JAGS MODEL #4

data.jags<-list('n.samp' = n.samp ,'y'= mompha,

'corolla' =corolla,

'flare' =flare,

'tube' =tube,

'herk' =herk,

'nectar' =nectar,

'section' = section,

'n.section' = n.section)

data.inits <- function (){

list (beta.corolla=rnorm(1),

beta.flare=rnorm(1),

beta.tube=rnorm(1),

beta.herk=rnorm(1),

beta.nectar=rnorm(1),

beta.section=rnorm(n.section),

sigma.y=runif(1))

}

data.parameters <- c ("beta.section","beta.corolla","beta.flare",

"beta.tube","beta.herk","beta.nectar",

"sigma.y")

data.jags.model <- jags.model(file ='/Users/taniajogesh/Dropbox/CBG Dimensions/Field Data/R files/CalyModel4.bug',

data = data.jags,

inits = data.inits,

n.chains = 4, n.adapt = 1000)

sdata.jags.model <- coda.samples(data.jags.model, data.parameters, n.iter = 100000)

round(summary(window(sdata.jags.model, start = 5000))$quantiles[,c(3, 1, 5)], 3)

gelman.diag(sdata.jags.model)$psrf

#==========================================

**model {**

**for (i in 1:n.samp){**

**y[i] ~ dnorm(y.hat[i], tau.y)**

**y.hat[i] <- beta.section[section[i]]**

**+ beta.corolla*corolla[i]**

**+ beta.flare*flare[i]**

**+ beta.tube*tube[i]**

**+ beta.herk*herk[i]**

**+ beta.nectar*nectar[i]**

**}**

**tau.y <- pow(sigma.y, -2)**

**sigma.y ~ dunif (0, 100)**

**beta.corolla~dnorm(0, 0.0001)**

**beta.flare~dnorm(0, 0.0001)**

**beta.tube~dnorm(0, 0.0001)**

**beta.herk~dnorm(0, 0.0001)**

**beta.nectar~dnorm(0, 0.0001)**

**for (j in 1:n.section) {**

**beta.section[j]~dnorm(mu.section, tau.section)**

**}**

**mu.section~ dnorm(0, 0.0001)**

**tau.section <- pow(sigma.section, -2)**

**sigma.section ~ dunif (0, 100)**

**}**

#==========================================

**# MODEL 5**

################## JAGS MODEL #5

K<- 6

x0<- rep(1,n.samp)

X<- cbind(x0,corolla, flare, tube, herk, nectar)

W<- diag(K)

U<-cbind(x0,species)

J<-n.species

L<-n.site

###########

data.jags<-list('N' = n.samp ,'y'= mompha, 'X' =X, 'species' = species,

'K' = K, 'J' = J,'W' = W)

data.inits <- function (){

list (B.raw=array(rnorm(J*K), c(J,K)),

mu.raw=rnorm(K),

sigma.y =runif(1),

Tau.B.raw = rwish(K+1, diag(K)),

xi=runif(K)

)

}

data.parameters <- c ("B","mu","sigma.y","sigma.B")#, "rho.B", " Tau.B.raw")

data.jags.model <- jags.model(file ='/Users/taniajogesh/Dropbox/CBG Dimensions/Field Data/R files/CalyModel5.bug',

data = data.jags,

inits = data.inits,

n.chains = 4, n.adapt = 1000)

sdata.jags.model <- coda.samples(data.jags.model, data.parameters, n.iter = 10000)

round(summary(window(sdata.jags.model, start = 5000))$quantiles[,c(3, 1, 5)], 3)

gelman.diag(sdata.jags.model)$psrf

#==========================================

**model {**

**for (i in 1:N){**

**y[i] ~ dnorm (y.hat[i], tau.y)**

**y.hat[i] <- inprod(B[species[i],],X[i,])**

**}**

**tau.y <- pow(sigma.y, -2)**

**sigma.y ~ dunif (0, 100)**

**for (j in 1:J){**

**for (k in 1:K){**

**B[j,k] <- xi[k]*B.raw[j,k]**

**}**

**B.raw[j,1:K] ~ dmnorm (mu.raw[], Tau.B.raw[,])**

**}**

**for (k in 1:K){**

**mu[k] <- xi[k]*mu.raw[k]**

**mu.raw[k] ~ dnorm (0, .0001)**

**xi[k] ~ dunif (0, 100)**

**}**

**Tau.B.raw[1:K,1:K] ~ dwish(W[,], df)**

**df <- K+1**

**Sigma.B.raw[1:K,1:K] <- inverse(Tau.B.raw[,])**

**for (k in 1:K){**

**for (k.prime in 1:K){**

**rho.B[k,k.prime] <- Sigma.B.raw[k,k.prime]/**

**sqrt(Sigma.B.raw[k,k]*Sigma.B.raw[k.prime,k.prime])**

**}**

**sigma.B[k] <- abs(xi[k])*sqrt(Sigma.B.raw[k,k])**

**}**

**}**

#==========================================

**# MODEL 6**

################## JAGS MODEL #6

K<- 6

x0<- rep(1,n.samp)

X<- cbind(x0,corolla, flare, tube, filament, nectar)

W<- diag(K)

J<-n.site

L<-n.species

###########

data.jags<-list('N' = n.samp ,'y'= mompha, 'X' =X, 'site' = site,

'K' = K, 'J' = J,'W' = W)

data.inits <- function (){

list (B.raw=array(rnorm(J*K), c(J,K)),

mu.raw=rnorm(K),

sigma.y =runif(1),

Tau.B.raw = rwish(K+1, diag(K)),

xi=runif(K)

)

}

data.parameters <- c ("B","mu","sigma.y","sigma.B")#, "rho.B", " Tau.B.raw")

data.jags.model <- jags.model(file ='/Users/taniajogesh/Dropbox/CBG Dimensions/Field Data/R files/CalyModel6.bug',

data = data.jags,

inits = data.inits,

n.chains = 4, n.adapt = 1000)

sdata.jags.model <- coda.samples(data.jags.model, data.parameters, n.iter = 10000)

round(summary(window(sdata.jags.model, start = 5000))$quantiles[,c(3, 1, 5)], 3)

gelman.diag(sdata.jags.model)$psrf

#==========================================

**model {**

**for (i in 1:N){**

**y[i] ~ dnorm (y.hat[i], tau.y)**

**y.hat[i] <- inprod(B[site[i],],X[i,])**

**}**

**tau.y <- pow(sigma.y, -2)**

**sigma.y ~ dunif (0, 100)**

**for (j in 1:J){**

**for (k in 1:K){**

**B[j,k] <- xi[k]*B.raw[j,k]**

**}**

**B.raw[j,1:K] ~ dmnorm (mu.raw[], Tau.B.raw[,])**

**}**

**for (k in 1:K){**

**mu[k] <- xi[k]*mu.raw[k]**

**mu.raw[k] ~ dnorm (0, .0001)**

**xi[k] ~ dunif (0, 100)**

**}**

**Tau.B.raw[1:K,1:K] ~ dwish(W[,], df)**

**df <- K+1**

**Sigma.B.raw[1:K,1:K] <- inverse(Tau.B.raw[,])**

**for (k in 1:K){**

**for (k.prime in 1:K){**

**rho.B[k,k.prime] <- Sigma.B.raw[k,k.prime]/**

**sqrt(Sigma.B.raw[k,k]*Sigma.B.raw[k.prime,k.prime])**

**}**

**sigma.B[k] <- abs(xi[k])*sqrt(Sigma.B.raw[k,k])**

**}**

**}**

#==========================================
